# Direct observation of NMR transverse relaxation in nanopatterned clusters of iron oxide particles

**DOI:** 10.1002/mrm.29898

**Published:** 2023-10-23

**Authors:** Ilhan Bok, Beth Rauch, Alireza Ashtiani, Aviad Hai

**Affiliations:** 1 Department of Biomedical Engineering, University of Wisconsin–Madison, Madison, Wisconsin, USA; 2 Department of Electrical and Computer Engineering, University of Wisconsin – Madison, Madison, Wisconsin, USA; 3 Wisconsin Institute for Translational Neuroengineering (WITNe), Madison, Wisconsin, USA; 4 Department of Medical Physics, University of Wisconsin – Madison, Madison, Wisconsin, USA

**Keywords:** cluster, FSEMS, MRI, nanoparticle, relaxation, transverse

## Abstract

**Purpose:**

We aim to verify predictions showing T_2_ relaxation rate of nanoparticle clusters and its dependence on spacing, size, geometry, and pulse sequence.

**Methods:**

We performed a laboratory validation study using nanopatterned arrays of iron oxide nanoparticles to precisely control cluster geometry and image diverse samples using a 4.7T MRI scanner with a T_2_-weighted fast spin-echo multislice sequence. We applied denoising and normalization to regions of interest and estimated relative R_2_ for each relevant nanoparticle array or nanocluster array. We determined significance using an unpaired two-tailed *t*-test or one-way analysis of variance and performed curve fitting.

**Results:**

We measured a density-dependent T_2_ effect ($p=8.9976\times 10^{-20}$, one-way analysis of variance) and insignificant effect of cluster anisotropy (p=0.5924, unpaired *t*-test) on T_2_ relaxation. We found negative quadratic relationships $\left(-0.0045{\left[log\;\tau_{D}\right]}^{2}-0.0655\left[log{\;\tau }_{D}\right]-2.7800\right)$ for single nanoparticles of varying sizes and for clusters $\left(-0.0045{\left[log{\;\tau }_{D}\right]}^{2}-0.0827\left[log\;\tau_{D}\right]-2.3249\right)$ for diffusional correlation time $\tau_{D}={r_{p}}^{2}/D$. Clusters show positive quadratic relationships for large $\left(3.8615\times 10^{-6}{\left[d_{pp}/r_{p}\right]}^{2}-9.3853\times 10^{-5}\left[d_{pp}/r_{p}\right]-2.0393\right)$ and exponential relationships for small $\left(-2.0050{\left[d_{pp}/r_{p}\right]}^{0.0010}\right)$ clusters. Calculated R_2_ peak values also align well with in silico predictions (7.85 × 10^−4^ ms compared with 1.47 × 10^−4^, 4.23 × 10^−4^, and 5.02 × 10^−4^ ms for single iron oxide nanoparticles, 7.88 × 10^−4^ ms compared with 5.24 × 10^−4^ ms for nanoparticle clusters).

**Conclusion:**

Our verification affirms longstanding in silico predictions and demonstrates aggregation-dependent behavior in agreement with previous Monte Carlo simulation studies.

## 1 | INTRODUCTION

T_2_ relaxation in MRI and readouts from other recently evolving imaging modalities such as magnetic particle imaging^1,2^ and optically detected MR,^3,4^ are all modulated by multiplexed, concentration-dependent features of magnetic tracers, sensors, and substrates.^2,4,5^ In clinical setting, superparamagnetic iron oxide nanoparticles (SPIONs) can specifically serve as the agent of choice for tissue contrast enhancement^6–8^ and perfusion-dependent structural readouts.^7–9^ SPIONs usually act as purely passive isotropic injectable agents, demonstrating an inherent ability to accumulate in different tissue types. Examples include hepatic lumen cells,^10–12^ splenic red pulp cells,^10,13^ gliosarcoma,^14^ and many more.^5^ These provide powerful diagnostic tools for detecting structural and tissue manifestationsofpathologiessuchascirrhosis,^10^ liver cancer,^10,11^ spleen cancer,^15^ and brain disorders.^16^ In addition to static image contrast enhancement, newly emerging responsive SPION-based sensors rely on specialized chemical coating to enable dynamic functional readouts of biophysical and biochemical components.^17–19^ Examples include SPIONs conjugated with calmodulin and its target peptides^20,21^ or C2 domains of synaptotagmin^22^ as calcium-responsive MRI contrast agents; engineered monoaminergic binding peptide domains for sensing neurotransmitters^23^; and more.^24–27^ Moreover, other types of injectable nanoparticles, nanostructures, and molecular probes offer aggregation-based sensing and modulation of electric fields^28–32^ and biochemicalprocesses^33–35^ with particular uses in neuroscience and neurology.^36^ The spatial distribution and related aggregation attributes of magnetic particles are important to both static and dynamic contrast enhancement, as the final 3D scaling factor, arrangement, and distribution of tracers can affect image quality, contrast, SNR, and sensitivity to analytes. This highlights the importance of precise prediction of SPION aggregation and corresponding MRI signal changes accompanying their nanoscale spatial organization.

Theoretical predictions of changes in relaxation rate (R_2_) due to SPION aggregation reveal a dependence on particle size, geometry, and anisotropy.^37–41^ Random walk simulations of water molecules diffusing in proximity to SPIONs suggest these nonlinearities also depend on scan parameters.^40,41^ Specifically, spin phase dispersion surrounding small nanoparticles are governed by classic outer sphere theory^37^ in what is termed the simple motional averaging regime. This effect plateaus and decreases for larger nanoparticles using spin-echo pulse sequences with large enough TE in what is termed the slow motion regime.^42^ For single nanoparticles of radius $r_{p}$ in medium with self-diffusion constant (D), the transition between these two regimes is represented by the diffusional correlation time $\left(\tau_{D}={r_{p}}^{2}/D\right)$ and is predicted to occur at a point inversely proportional to the angular frequency shift at the particle surface $\left(\Delta \omega_{r}\right)$.^43^ More recent models attempt to describe this relationship for clustered SPIONs in the context of aggregation-based MRI sensing^41^ reporting peak R_2_ transition value between the motional averaging regime and slow motion regime of 85.59 s^−1^ at $\tau_{D}=5.02\times 10^{-4}\;ms$ for single particles/dots and 87.31 s^−1^ at $\tau_{D}=5.24\times 10^{-4}\;ms$ for 3D isotropic clusters. Chemical exchange models of diffusion^38^ demonstrated comparable peak R_2_ value of 0.45 s^−1^ for echo spacing $\tau_{CP}=0.5\;ms$ and $\Delta \omega_{r}=472\;rad/s$, at $\tau_{D}=0.15\;ms\;\left(\tau_{D}/\tau_{CP}=1.97\right)$ with a volume fraction (v) of 0.005: Normalizing by v yields $\tau_{D}=1.47\times 10^{-4}$. The chemical exchange model posits that water molecules either diffuse past iron oxide nanoparticles (“outer sphere”) or contact and bind them (“inner sphere”) and that flux between these two states influences diffusion-related T_2_ relaxation behavior. A few measurements have supported these studies indirectly,^44–48^ but a piecewise ground-truth experimental validation of this effect has not yet been performed.

Here we use precise nano-scale lithography of SPION clusters to provide direct experimental observation of the concept of significant alterations in R_2_ due to aggregation. By extracting R_2_ values from MRI measurements of nanofabricated arrays of SPION clusters, we demonstrate peak diffusion time correlating with model predictions, with peak $\tau_{D}$ measured at 7.85 × 10^−4^ ms for single particles. Peak $\tau_{D}$ for isotropic 2D clusters was 7.88 × 10^−4^ ms and similarly correlated. Our results present a first validation and agreement with long-standing theoretical predictions of the behavior of iron oxide nanoparticles and provide a novel protocol with broad implications on the analyses and development of MRI contrast agents and aggregation-based sensors and modulators.

## 2 | METHODS

### 2.1 | Finite element analyses of magnetic fields

Magnetic field profiles of SPION clusters were simulated using COMSOL Multiphysics 6.0 (COMSOL Inc., Stockholm, Sweden). The Magnetic Fields (mf) module was used to emulate nanoparticle arrays under a 4.7T B_0_ bias field applied along the y-direction within a hexagonal simulation arena with periodic boundary condition. The Ampere’s Law boundary condition with a B-H curve magnetization model was applied on low carbon steel magnetite particles (COMSOL Materials Library) surrounded by CSF (relative electrical permittivity $\varepsilon_{r}=81.2$, electrical conductivity σ=4.8S/m). A Job Configurations node was used to perform a parametric sweep of interparticle distance $\left(d_{pp}\right)$ to particle radius $\left(r_{p}\right)$ ratio $\left(d_{pp}/r_{p}\right)$, ranging from 3 to 40 in 0.1 increments. The resultant magnetic field values were processed using *Python 3.7* as follows: Values were weighted by mesh element size within a region of radius 12*dpp at the center, left, or right region of the nanoparticle cluster, and an origin-centered circle with radius $2*d_{pp}$ was used to define the FOV of the cluster. Statistical traces were further processed and visualized using *MATLAB* R2022a (MathWorks, Inc., Natick, MA, USA). A moving average centered between the current and previous elements with a sliding window of four frames was used to smooth the mean and SD for all field traces against $d_{pp}/r_{p}$. Averages were truncated at endpoints where there were not enough elements to fill the moving mean window.

### 2.2 | Nanostructure fabrication

**FIGURE 1:**
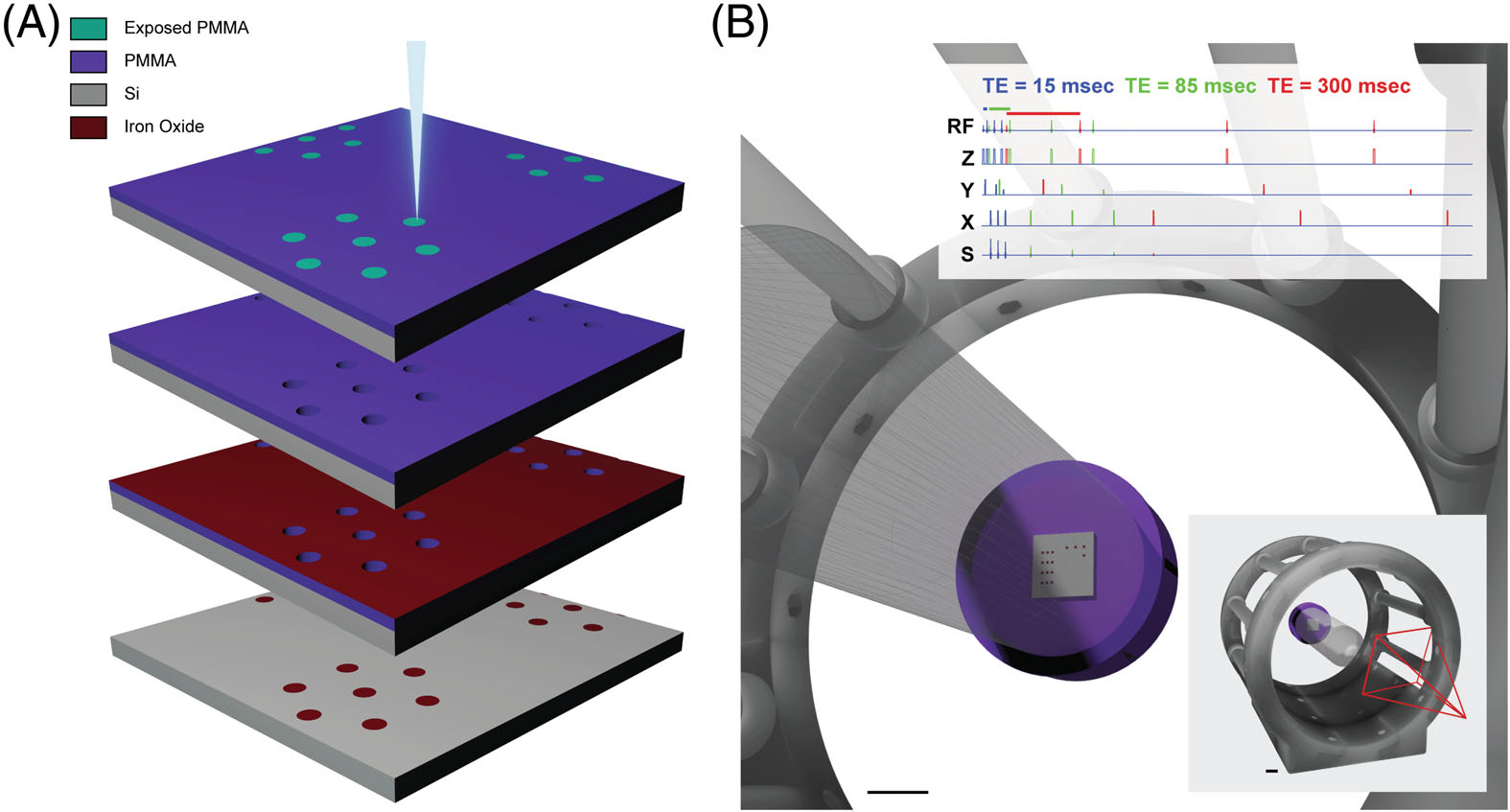
Iron oxide nanoparticle cluster fabrication and MR scan schematic. (A, from top to bottom) A layer of poly methyl-methacrylate is spin-coated onto a silicon substrate and patterned using electron beam lithography. The layer is then developed using a 1:3 mixture of methyl isobutyl ketone: isopropanol, coated with an ultrathin layer of iron oxide using electron beam metal evaporation, then lifted off using room-temperature acetone with gentle agitation. (B) A scanning procedure schematic showing the iron oxide chip attached to the inside of a conical tube, placed into a birdcage coil (scale bar = 10 mm) and scanned using multislice fast spin echo (upper right) with variable TE. Values from 15 to 300 ms in 15 ms increments were recorded. An image using a TE of 85 ms was chosen for further analysis (see Figure 4). The full configuration is shown on the bottom right, with the perspective seen in the full panel shown as a red prism (scale bar = 10 mm).

We used electron beam nanolithography to create nanopatterned iron oxide arrays (Figure 1A). A silicon N-type phosphorus doped <100> 1–10Ω·cm 380 μm single-side polished wafer (Cat. Number 695; UniversityWafer, Boston, MA, USA) was spin-coated with poly methyl-methacrylate 495 A4 photoresist (Kayaku Advanced Materials Inc., Westborough, MA, USA) at 4000rpm for 45 s. After verifying a film thickness of 185nm (F20 Reflectometer; Filmetrics, Inc., San Diego, CA, USA), the wafer sample was baked on a hotplate at 180°C for 90 s. The baked wafer was diced into 10 × 10 mm chips and patterned using an Elionix ELS G-100 electron beam lithography system (Elionix Inc., Tokyo, Japan) using the parameters given in Table 1. Samples were developed at room temperature in 1:3 methyl isobutyl ketone (Cat. Number M2131; Thermo Fisher Scientific Inc., Waltham, MA, USA):isopropanol (MIBK:IPA) for 60 s and rinsed first with IPA, then with deionized (DI) water. After inspecting the consistency of developed samples (Figure S1) using a Zeiss LEO 1530–1 field emission scanning electron microscope (SEM; Carl Zeiss AG, Oberkochen, Germany), a custom-built electron beam metal evaporator was used to deposit a 30-nm layer of iron oxide at 0.2Å/s at room temperature (~20°C) and relative humidity of 23% or less. Samples were lifted off in room temperature acetone with gentle agitation to prevent re-adhesion of iron. Completed samples were rinsed first with IPA, then with DI water. Structures were visually inspected for cleanliness and consistency after each processing step using an Olympus BX51WI Upright Fluorescent Microscope (Olympus, Tokyo, Japan).

### 2.3 | Fast spin-echo multislice MRI T_2_ scans

**FIGURE 2:**
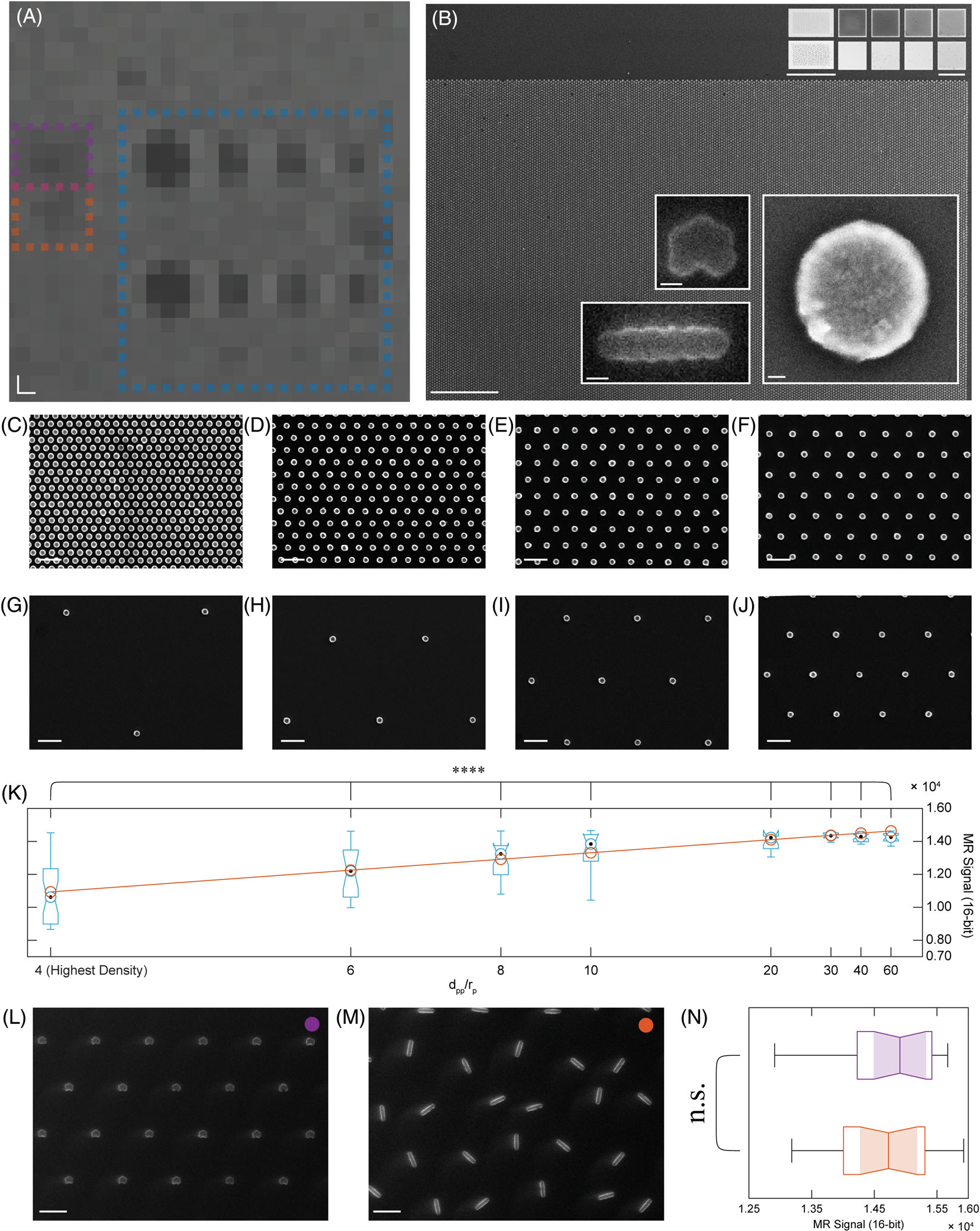
Magnetic resonance (MR) and scanning electron microscopy (SEM) images of nanopatterned iron oxide arrays reveal density dependence and a non-significant effect of anisotropy on R_2_. (A) T_2_-weighted fast spin-echo multislice MR images reveal a density-dependent response to 100 × 100 μm^2^ nanopatterned iron oxide arrays (diameter = 200 nm, scale bar = 200 × 200 μm). Blue, violet, and orange boxes denote regions corresponding to (C)-(J), (L), and (M), respectively. (B) Viewing the nanopatterned iron oxide arrays under SEM (main panel, $d_{pp}/r_{p}=4$, scale bar = 10 μm) and optical microscopy (upper insets, left column scale bar = 50 μm, other columns scale bar = 100 μm) shows density-dependent darkening as well as differing anisotropy for chains (bottom-left inset, scale bar = 200 nm) and clusters (upper-left inset, scale bar = 200 nm). A single-nanoparticle SEM shows consistency and uniformity when approaching single-atom resolution (right inset, scale bar = 30 nm). SEM scans of interparticle spacing to radius $\left(d_{pp}/r_{p}\right)$ ratios of 4(C), 6(D), 8(E), 10(F), 60(G), 40(H), 30(I), and 20(J) in the same order as (A) and (B) are shown below (scale bar = 1 μm). (K) Box plot of relative MR signal intensity versus $d_{pp}/r_{p}$. Black dots denote the median; notches denote bounds of statistical significance; and whiskers denote outlier thresholds (from Q1-W×[Q3-Q1] to Q3+W×[Q3-Q1] where W=1.5). The orange line and data points represent a linear curve fit to the medians of each $d_{pp}/r_{p}\left(m=1.5850\times 10^{4}\;[MR\right.\;signal]/{\left[d_{pp}/r_{p}\right]}^{-1}$, $b=2.7895\times 10^{4}\;\left[MR\right.\;signal]$; $p=8.9976\times 10^{-20}$, ****p<0.0001, one-way analysis of variance, R_2_ = 0.9442). SEM images of nanoparticle clusters (L) and randomly oriented nanochains (M) (scale bar = 2 μm).(N) Pixel intensity analysis of both regions reveals no significant effect of anisotropy on R_2_ (p=0.5924, unpaired *t*-test).

Nanofabricated samples were epoxied (Cat. Number 078143–14210; R.S. Hughes Co., Inc., Sunnyvale, CA, USA) with the patterned side facing up on a cylindrical high density polyethylene surface (radius = 10 mm, height = 10 mm), which was itself epoxied into a 50-mL centrifuge tube cap, and the entire phantom tube was filled with reagent-grade DI water. Images were acquired using fast spin-echo multislice T_2_-weighted scans (Table 2) on an Agilent 4.7T horizontal-bore MRI/MRS system housing a 72-mm inner diameter Agilent/Varian quad birdcage volume coil (#S190888200 108/38 1H 200 MHz; Varian Medical Systems, Inc., Palo Alto, CA, USA) (Figure 1B). For density-dependent and anisotropy-dependent samples (Figure 2), we used TE = 68 ms, TR = 4000 ms, and a voxel size of 78.1 × 79.4 μm with a slice thickness of 700 μm and *N* = 10 averages. For arrays of varying nanoparticle size and nanoparticle clusters, scans used TE = 85 ms, TR = 5000 ms, and a voxel size of 70.3 × 71.4 μm with a slice thickness of 400 μm and *N* = 12 averages.

### 2.4 Quantifications of density-dependent MR intensity trends

Sixteen-bit DICOM (Digital Imaging and Communications in Medicine) MR images were thresholded below a value of 1.47 × 10^4^. For density-dependent DICOM images, *ImageJ* (National Institutes of Health, Bethesda, Maryland, USA) was used for background correction assuming a light background and a rolling ball radius of 50 px. A 4 px× 4 px selection window was applied to each density. Raw optical images were thresholded at 6.02 × 10^4^, and a polygonal selection was manually applied to each region of interest (ROI). For both image analyses, a least-squares linear fit of iron oxide nanoparticle concentration to median pixel value was used to determine R_2_.

### 2.5 | Quantifications of relative R_2_ trends

To determine R_2_ for each ROI, *Python* 3.7 was used to curve fit a Gaussian probability density function to a reference histogram of background pixel intensities according to:

(1) $$w=normalpdf\left(min\left(\mu_{B},S\right),\sigma_{B}\right)$$

where S is the raw MR signal matrix; $\mu_{B}$ is the mean background brightness; $\sigma_{B}$ is the SD of background brightness; and normalpdf is probability density function of the Gaussian distribution. We then shifted this mean by a constant value for each ROI to compensate for background field inhomogeneities before calculating R_2_ values. The resultant pixel weight vectors (w) were used to find T_2_ values for each voxel ($S_{T2}$) using:

(2) $$S_{T2}=\left(1-w\sigma_{B}\sqrt{2\pi }\right)\;*\;S$$

The output was processed by a Gaussian filter to remove noise (see Table 3 for σ parameter and resultant SNR values for each ROI type) and estimated relative R_2_ with:

(3) $$\mu_{R2}=mean\left(log\;{\left(S_{T2}\right)}^{-1}\right)$$

where $\mu_{R2}$ is the mean relative R_2_ over each ROI normalized to plots of simulated relaxivity changes with minor variations between the corresponding plots.^1^

## 3 | RESULTS

### 3.1 | T_2_ measurements of iron oxide nanoparticle arrays reveal density-dependent and anisotropy-independent behavior

T_2_-weighted fast spin-echo multislice MR images of 200-nm nanopatterned iron oxide particles affirm a density-dependent (particles per unit area) response in 100 × 100 μm^2^ arrays and nonsignificant effect of structure anisotropy (Figure 2A). SEMs (Figure 2B, main panel and lower insets) and optical microscopy (Figure 2B, upper insets) of nanopatterned particles verify consistent linear density-dependent signal decrease (Figure S2) and variable anisotropy for nanoparticle chains and clusters (Figure 2B, upper left, optical; bottom left, SEM). A single-nanoparticle SEM shows structural uniformity when at near single-atom resolution (Figure 2B, lower right inset). SEMs of interparticle spacing to radius $\left(d_{pp}/r_{p}\right)$ ratios of 4, 6, 8, 10, 60, 40, 30, and 20 are shown in Figure 2C–J, respectively, and in the same order as Figure 2A,B. Comparing MRI voxel intensity versus ${\left(d_{pp}/r_{p}\right)}^{-1}$ reveals a density-dependent response (Figure 2K). Shown in Figure 2K are black dots denoting the median, notches denoting bounds of statistical significance, and whiskers denoting outlier thresholds from Q1–1.5×(Q3–Q1)toQ3+1.5×(Q3–Q1), where Q1 is the first quartile or 25th percentile, Q3 is the third quartile or 75th percentile, (Q3-Q1) is the interquartile range, and 1.5×(Q3-Q1) is the outlier cutoff threshold. The orange curve in Figure 2K and corresponding data points represent a linear curve fit to the medians of each ${\left(d_{pp}/r_{p}\right)}^{-1}\;\left(m=1.5850\times 10^{4}\;(MR)\right.\;signal)/{\left(d_{pp}/r_{p}\right)}^{-1}$, $b=2.7895\times 10^{4}$ (MR signal) ($p=8.9976\times 10^{-20}$, ****=p<0.0001, one-way analysis of variance). Minor variations in brightness exist at lower densities ($d_{pp}/r_{p}$ ranging between 30 and 60), independent of background gradient correction. SEM images of compact nanoparticle clusters and randomly oriented and positioned nanoparticle chains (Figure 2L,M, respectively) confirm the consistency of our cluster fabrication method. MRI signal brightness of both regions reveal no significant effect of anisotropy on T_2_ decay (Figure 2N; p=0.5924, unpaired *t*-test). Based on these verifications of uniformly dense arrays of single nanoparticles, we turned to finite element analysis to predict the effect of nonuniform aggregation on magnetic fields.

### 3.2 | Finite element analysis of hexagonal nanoparticle clusters affirms proximity-dependent field enhancement

**FIGURE 3:**
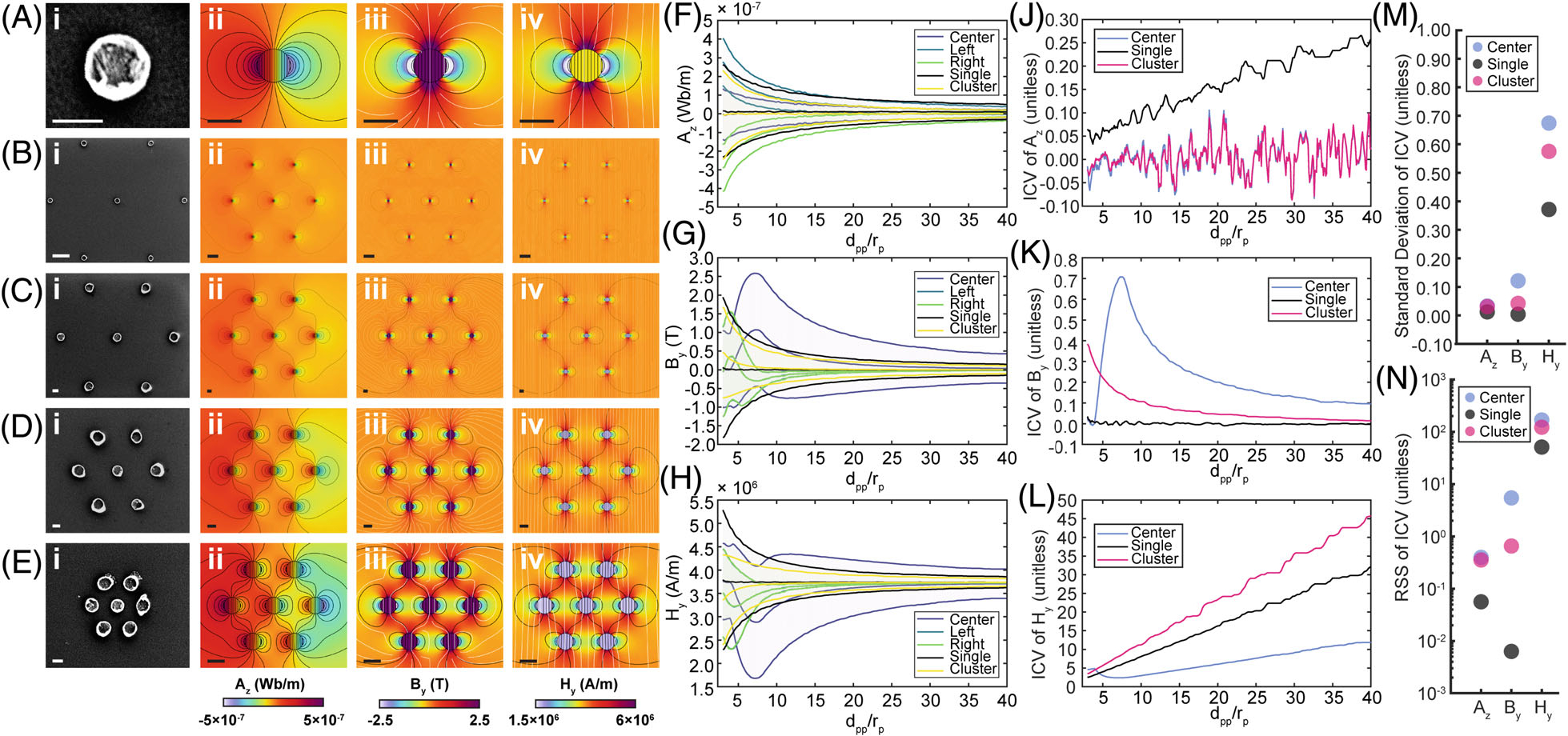
Finite element analysis demonstrates highly diverse magnetic fields in proximity to nanoparticle clusters compared with single nanoparticles. Data for nanoparticles (A), nanoparticle clusters with $d_{pp}/r_{p}=40$ (B), $d_{pp}/r_{p}=20$ (C), $d_{pp}/r_{p}=10$ (D), and $d_{pp}/r_{p}=5$ (E) (nanoparticle diameter = 200 nm; scale bar = 200 nm except [B], where scale bar = 1 μm). SEM (A[i]-E[i]), magnetic vector potential z-component $A_{z}$ (A[ii]-E[ii]), magnetic flux density y-component $B_{y}$ (A[iii]-E[iii]), magnetic field intensity y-component $H_{y}$ (A[iv]-E[iv]) of various $d_{pp}/r_{p}$. Corresponding running-average (n=4) mean and SD of a nanoparticle and center, left, right, and entire cluster are shown for $A_{z}(F)$, $B_{y}(G)$, and $H_{y}(H)$. Changes in inverse coefficient of variation (ICV) for varying interparticle distance to particle radius ([F]–[H]). The ICV, defined as the mean divided by the SD of field values around particles in the simulation arena, for magnetic vector potential z-component $A_{z}(J)$, magnetic flux density y-component $B_{y}(K)$, and magnetic field intensity y-component $H_{y}(L)$ of $d_{pp}/r_{p}$ ranging from 3.0 to 40.0 in increments of 0.1. Although the ICV increases relatively linearly for single particles, changes are more stochastic or highly nonlinear for the entire cluster, particularly the nanoparticle at the center of the cluster. Comparing the residual sum of squares (RSS) (M) and the adjusted SD of the ICV versus magnetic field type (N) shows that single nanoparticles are less variable than clusters and center nanoparticles.

We used finite element analysis to quantify B_0_-induced magnetization in single nanoparticles (Figure 3A), and clusters of nanoparticles with $d_{pp}/r_{p}=40$ (Figure 3B), $d_{pp}/r_{p}=20$ (Figure 3C), $d_{pp}/r_{p}=10$ (Figure 3D), and $d_{pp}/r_{p}=5$ (Figure 3E). SEM reference images (Figure 3A[i]–E[i]) show proof of principle structures for simulations of magnetic vector potential (Figure 3A[ii]–E[ii]), magnetic flux density (Figure 3A[iii]–E[iii]), and magnetic field intensity (Figure 3A[iv]–E[iv]) of varying cluster values of $d_{pp}/r_{p}$. Corresponding running-average mean and SD of fields across a single nanoparticle (Figure 3F–H, black curves), from the center, left, and right regions of a single nanoparticle (Figure 3F–H, purple, blue, green curves, respectively), and across the entire cluster (Figure 3F–H, yellow) are shown for magnetic vector potential z-component ($A_{z}$, Figure 3F), magnetic flux density y-component ($B_{y}$, Figure 3G), and magnetic field intensity y-component ($H_{y}$, Figure 3H). The inverse coefficient of variation (ICV), defined as the mean field amplitude divided by the SD (μ/σ) surrounding nanoparticles in the simulation arena (Figure 3J–L), shows greater variability for clustered nanoparticles compared with single nanoparticles under the same simulation conditions, demonstrating a clear aggregation-related effect. Specifically, the residual sum of squares (RSS) for single nanoparticles for $A_{z}$, $B_{y}$, and $H_{y}$ are 0.0561, 0.0063, and 51.0562, whereas for the center nanoparticle in a cluster they are $0.3968\;\left(7.0733\;\times \right.\;\left.{RSS}_{\text{single}}\right)$, 5.4398 (862.6164), and 168.2382 (3.2952), and for the whole cluster the values are 0.3537 (6.3044), 0.6542 (103.7460), and 122.3860 (2.3971) (Figure 3M). The larger RSS for clusters affirms that iron oxide nanoparticle clusters exhibit highly variable fields compared with single nanoparticles. Although ICV increases relatively linearly for single nanoparticles, changes are more stochastic or highly nonlinear for the entire cluster and single nanoparticles at the cluster center. Subtracting the line of best fit yields SDs for $A_{z}$, $B_{y}$, and $H_{y}$ of 0.0123, 0.0041, and 0.3715 for single nanoparticles, $0.0327\;\left(2.6596\times \sigma_{\text{single}}\right),0.1213$ (29.3703), and 0.6743 (1.8153) for center nanoparticles, and 0.0309 (2.5109), 0.0421 (10.1856), and 0.5751 (1.5483) for the whole cluster (Figure 3N). Of note are ICVs for magnetic flux density (Figure 3K), which fluctuate from −0.0099 at $d_{pp}/r_{p}=20.9$ to 0.0345 at $d_{pp}/r_{p}=3.0$ for isolated single iron oxide nanoparticles compared with fluctuations from −0.0077 at $d_{pp}/r_{p}=3.6$ to 0.7086 at $d_{pp}/r_{p}=7.3$ for center iron oxide nanoparticles. These simulations therefore predict that aggregation of nanoparticles produces fields that show asymptotic behavior with increasing $d_{pp}/r_{p}$ and are spatially diverse compared with those of single nanoparticles.

### 3.3 | MR scan-derived R_2_ trends agree with Monte Carlo simulations

**FIGURE 4:**
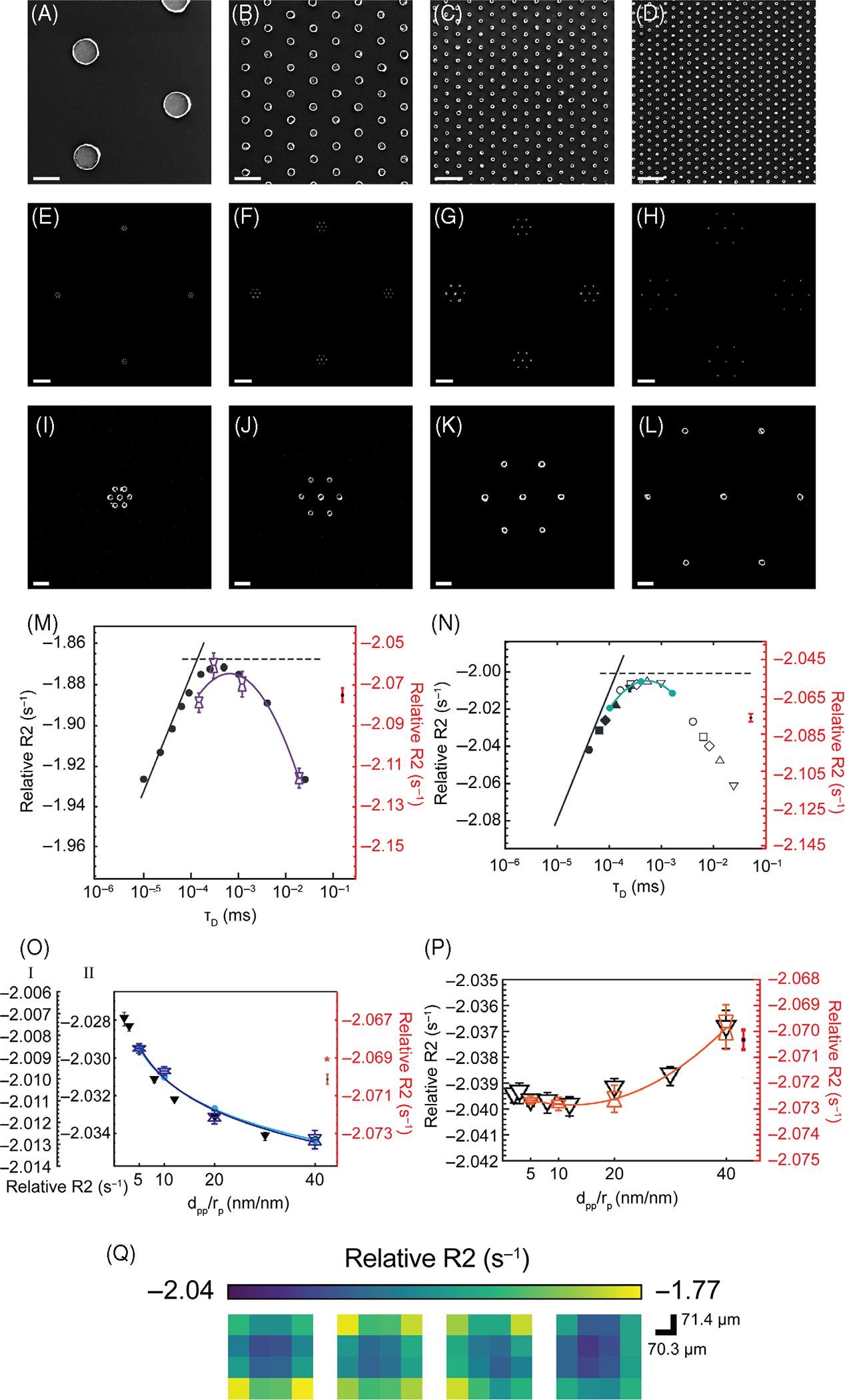
Scanning electron microscope (SEM) images and corresponding MR R_2_ plots of signal and noise show trends in agreement with previous Monte Carlo simulations. Shown are SEMs of different nanopatterned iron oxide arrays with $d_{pp}/r_{p}=10$ of sizes of 800nm (A), 200nm (B), 100nm (C), and 70nm (D) (scale bar = 1 μm). Below are SEMs of four representative nanoparticle clusters of $r_{p}=50\;nm$ and $d_{pp}/r_{p}=5$ (E), $d_{pp}/r_{p}=10$ (F), $d_{pp}/r_{p}=20$ (G), and $d_{pp}/r_{p}=40$ (H) (scale bar = 2 μm), corresponding to magnified panels (I–L, scale bar = 400nm). Relative T_2_ relaxation rates (R_2_) (left axis) and noise levels (right axis) normalized to previous simulations show agreement in diffusional correlation time $\tau_{D}={r_{p}}^{2}/D$ for single nanoparticles (M) (A–D) and nanoparticle clusters of nanoparticles (N) (E, inset panel I). (O) Relative R_2_ values from nanoparticle clusters of small nanoparticles ($r_{p}=35\;nm$ or 50nm as in [E]–[H] [inset panels (I)–(L)]), where I corresponds to raw data (light blue); II corresponds to Gaussian blurred data (navy blue); and * denotes noise level after Gaussian blurring. (P) Large nanoparticles ($r_{p}=400\;nm$ patterned like panels [E]–[H] [I-1], size differs) also show R_2_ trends versus $d_{pp}/r_{p}$ in agreement with Monte Carlo simulations. When present, lines denote the median; notches denote bounds of statistical significance; and whiskers denote outlier thresholds from Q1-1.5×(Q3-Q1)toQ3+1.5×(Q3-Q1), where Q1 is the first quartile or 25th percentile; Q3 is the third quartile or 75th percentile; (Q3-Q1) is the interquartile range; and 1.5×(Q3-Q1) is the outlier cutoff threshold. (Q) The corresponding MRI regions of interest for (M) after conversion to R_2_.

To corroborate nanoparticle aggregation–dependent and nanoparticle size–dependent field effects predicted in theory, we performed MRI of varying nanopatterned iron oxide cluster arrays (Figure 4). SEMs of constant $d_{pp}/r_{p}=10$ and variable nanoparticle sizes of 800nm (Figure 4A), 200nm (Figure 4B), 100nm (Figure 4C), and 70nm (Figure 4D) demonstrate consistent uniformity of nanoparticles and nanoparticle clusters (Figure 4A–L). Four representative nanoparticle clusters (and corresponding zoomed insets) with nanoparticle diameter of 100nm and $d_{pp}/r_{p}=5$ (Figure 4E,J), $d_{pp}/r_{p}=10$ (Figure 4F,J), $d_{pp}/r_{p}=20$ (Figure 4G,K), and $d_{pp}/r_{p}=40$ (Figure 4H,L) are shown. Our R_2_ analyses show agreement in diffusional correlation time $\tau_{D}=r_{p}^{2}/D$ for single nanoparticles (Figure 4M; corresponding SEMs in Figure 4A–D) and nanoparticle clusters (Figure 4N; see Figures 3E and 4E) and $d_{pp}/r_{p}$ for clusters of small nanoparticles ($r_{p}=35\;nm$ or 50nm; Figure 4O; see Figure 4E–H) and clusters of large nanoparticles ($r_{p}=400\;nm$; Figure 4P; see Figure 3B–E). When present, lines denote the median; notches denote bounds of statistical significance; and whiskers denote outlier thresholds from Q1–1.5×(Q3–Q1)toQ3+1.5×(Q3–Q1), where Q1 is the first quartile or 25th percentile, Q3 is the third quartile or 75th percentile, (Q3–Q1) is the interquartile range, and 1.5×(Q3-Q1) is the outlier cutoff threshold. Shown in Figure 4Q are corresponding MRI ROIs for panel (m) after conversion to R_2_. We found negative quadratic relationships $\left(-0.0045{\left[log{\;\tau }_{D}\right]}^{2}-0.0655[log\;\;\tau_{D}]-2.7800\right)$ (Figure 4M) for single nanoparticles of varying sizes and for clusters $\left(-0.0045{\left[log\;\tau_{D}\right]}^{2}-0.0827[log\;\;\tau_{D}]-2.3249\right)$ (Figure 4N) for varying diffusional correlation time $\tau_{D}=r_{p}^{2}/D$. Small clusters show changes smaller than the background noise level, which we fit quadratically $(3.2046\times 10^{-6}\;{\left[d_{pp}/r_{p}\right]}^{2}-2.6204\times 10^{-4}\left.\left[d_{pp}/r_{p}\right]-2.0075\right)$, inverse quadratically $\left(-7.9436\times 10^{-7}{\left[d_{pp}/r_{p}\right]}^{-2}+6.4888\times 10^{-5}{\left[d_{pp}/r_{p}\right]}^{-1}-0.4981\right)$ and exponentially ($-2.0050{\left[d_{pp}/r_{p}\right]}^{0.0010}$) (Figure 4O). We applied Gaussian filtering to reduce noise in this ROI and determined that the percent change in signal remained consistent (−0.2097% to -0.2382% [Figure 4O]; see Table 3 for SNR values). We additionally found that large clusters show positive quadratic relationships well above noise level ($3.8615\times 10^{-6}{\left[d_{pp}/r_{p}\right]}^{2}-9.3853\times 10^{-5}\left[d_{pp}/r_{p}\right]-2.0393$) (Figure 4P) in agreement with previous Monte Carlo simulation studies.^40,41^

## 4 | DISCUSSION AND CONCLUSIONS

Here we leveraged nano-scale lithography to precisely pattern clusters of iron oxide nanoparticles and quantify their R_2_ using fast spin-echo multislice MR scan data. We found nonlinear polynomial R_2_ dependence for both single and diversely clustered iron oxide dots with varying interparticle distance and particle radius. Furthermore, we reported an exponential aggregation-dependent relationship between interparticle distance and particle radius, verified by nonlinear least squares trust region reflective fit strategy. A 2D array of SPIONs situated on a planar silicon surface also primarily affects field perturbations for water molecules diffusing near the plane of the nanoparticle cluster array, which may help explain relatively low SNR for exponentially fitted small nanoparticles without Gaussian filtering. To characterize magnetic field behavior for a continuum of varying interparticle spacings for nanoparticle clusters, we analyzed magnetic field maps from our finite element nanoparticle cluster model and found increasing variability quantified by both ICV and RSS with decreasing interparticle distance to particle radius ratio $\left(d_{pp}/r_{p}\right)$. We note that while we investigated T_2_ relaxation effects for nanoparticles with radius as small as 35nm, some clinically used SPIONs can be as small as 4nm.^6^ Earlier theoretical work shows that MNPs below 10nm in diameter are expected to continue trends predicted by microscopic outer sphere theory.^37^ Extrapolating our experimental results yields a similar decline in the form of relative R_2_ of −2.006s^−1^ for 10-nm nanoparticles and −2.214s^−1^ for clusters, correlating with theoretical predictions and corresponding to the quadratic nature of diffusion distances versus time, where by water molecules are expected to diffuse past small particles (<10nm) more than an order of magnitude faster than 35-nm particles. Nonetheless, smaller particle sizes usually result in more efficient tissue clearance, and the effect on diffusing water molecules described here coincides with the overall applicability of biomedically relevant single nm SPIONs.^49,50^ Some discrepancies could be a result of nano-scale and pico-scale variations in fabricated structures compared with chemical synthesis of SPIONs. The ability to determine precise peak R_2_ values by nanopatterning diverse cluster topologies can drive the design of new sensor technologies for MRI. Our fabrication protocol could be expanded for patterning other particle material compositions and even patterning of widely used molecular agents. Broadening the scope of both native and modified nanoparticles for magnetic particle imaging,^51^ optically detected MR,^52^ and fluorescent imaging^53^ could help optimize static and dynamic image contrast, SNR, and sensitivity to analytes without sacrificing biocompatibility or resolution. Future experiments will consist of nanofabricating 2D and 3D array combinations composed of self-assembling modules with additional geometries to confirm that our work extends to three dimensions and performing multimodal magnetic imaging of array samples implanted in vivo. In conclusion, our analyses agree with and contribute further understanding into iron oxide nanoparticle aggregation-dependent field behavior observed in theoretical predictions. Our results lay a robust and adaptable foundation for the design and development of nanometer-scale and micrometer-scale contrast agents and probes for MRI and related modalities.

## Supplementary Material

Supplementary

## Figures and Tables

**TABLE 1:** Parameters used for electron beam lithography patterning of Si/PMMA substrate.

Array type	Density/anisotropy	Size/cluster
Field size (μm)	250	250
Dot number	500 000	500 000
Exposure (μC/cm^2^)	800	(800, 1600, 2400)
Feed pitch	10	10
Scan pitch	10	10
Beam current (nA)	2	10
Exposure time (μs/dot)	0.10	(0.02, 0.04, 0.06)
Matrix size (μm × μm)	100.0 × 100.0 (density)	100.0 × 100.0
	122.5 × 70.0 (anisotropy)	

**TABLE 2:** Parameters used for MR scans of iron oxide nanoparticle arrays.

Scan parameters	Density and anisotropy arrays	Size and spacing arrays
Pulse sequence	FSEMS	FSEMS
Field strength (T)	4.7	4.7
TE (ms)	68	85
TR (ms)	4000	5000
Voxel size (μm × μm)	78.1 × 79.4	70.3 × 71.4
Slice thickness (μm)	700	400
Number of averages	10	12

**TABLE 3:** Gaussian blurring parameters and corresponding SNR values for R_2_ plots in Figure 4.

Analysis type	Region-of-interest size	Gaussian kernel *σ*	μ noise (s^−1^)	σ noise (s^−1^)	SNR (dB)
Single	3 × 3	1	−2.071	1.071 × 10^−3^	44.530
Cluster	2 × 2	0	−2.076	2.173 × 10^−3^	24.983
Small clusters	2 × 4	0	−2.074	3.349 × 10^−3^	25.134
Small clusters Gaussian blurring	2 × 2	2	−2.070	2.652 × 10^−4^	43.198
Large clusters	2 × 3	2	−2.070	3.800 × 10^−4^	37.429

## Footnotes

1 Resources for quantifying R2 relaxation from 2D clusters and arrays of SPIONs are available at https://github.com/hailab-uw/2D_SPIO_R2
