# Supplementary material for: Direct observation of NMR transverse relaxation in nanopatterned clusters of iron oxide particles
Source: Magn Reson Med. Author manuscript; Available in PMC 2025 Feb 1. (PMC11489851; doi:10.1002/mrm.29898)
Supplement: Supplementary file 1 [file NIHMS2025711-supplement-Supplementary.rtf]

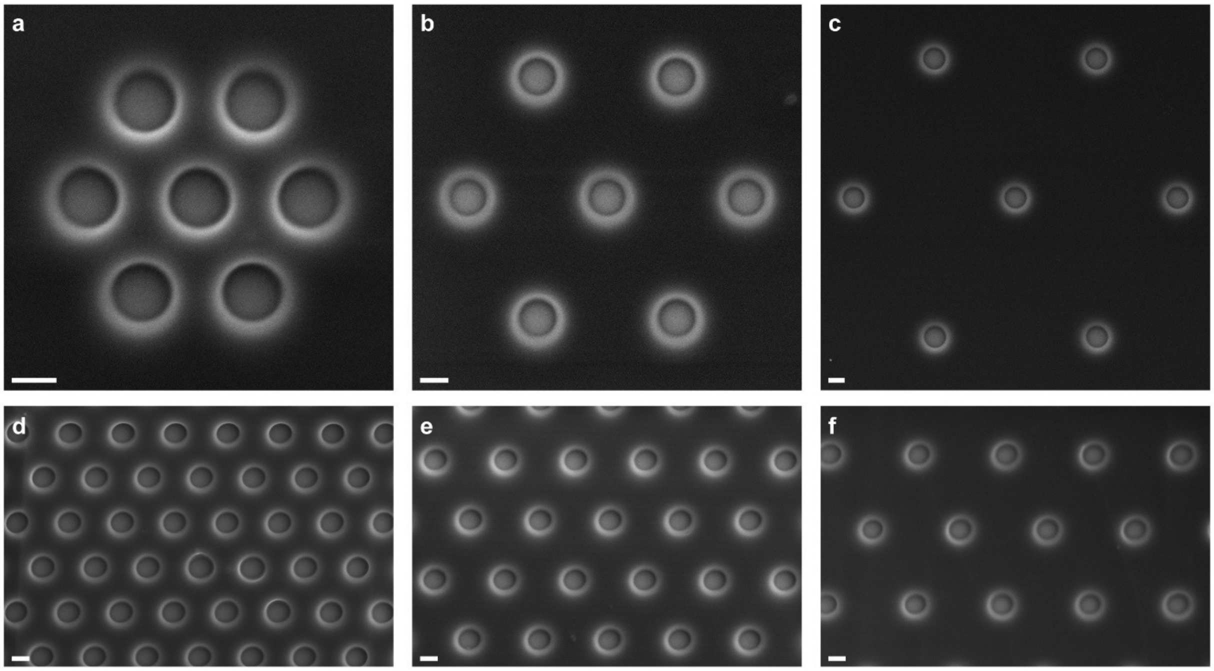

Figure S1: Scanning electron microscope (SEM) images of developed poly methyl-methacrylate before iron oxide deposition. Shown are SEMs of representative hexagonal nanovoids patterned on poly methyl-methacrylate with (a) d_{pp}/r_p = 5, (b) d_{pp}/r_p = 10, and (c) d_{pp}/r_p = 20, and nanovoids with spacing (d) d_{pp}/r_p = 6, (e) d_{pp}/r_p = 8, and (f) d_{pp}/r_p = 10 (scale bars = 200 nm).

Figure S2: Linear fit of iron oxide nanostructure array optical image intensity. Box plot of optical image intensity (see square insets in upper right of Fig. 2b) versus d_{pp}/r_p. Black dots denote the median, notches (present but not visible) denote bounds of statistical significance, and whiskers denote outlier thresholds (from $Q1 - W \times (Q3 - Q1)$ to $Q3 + W \times (Q3 - Q1)$ where $W = 1.5$). The orange line and data points represent a linear curve fit to the medians of each d_{pp}/r_p ($m = 1.6475 \times 10^5$ (Optical intensity)/$(d_{pp}/r_p)^{-1}$, $b = 6.0603 \times 10^4$ (Optical intensity); $p = 0$, one-way ANOVA, R^2 = 0.8915).
